# Inverse molecular docking reveals a novel function of thymol: Inhibition of fat deposition induced by high‐dose glucose in *Caenorhabditis elegans*

**DOI:** 10.1002/fsn3.2392

**Published:** 2021-06-11

**Authors:** Fangfang Ban, Liangbin Hu, Xiao‐Hui Zhou, Yanyan Zhao, Haizhen Mo, Hongbo Li, Wei Zhou

**Affiliations:** ^1^ School of Food Science Henan Institute of Science and Technology Xinxiang China; ^2^ Department of Food and Bioengineering Shaanxi University of Science & Technology Shaanxi China; ^3^ Department of Pathobiology & Veterinary Science University of Connecticut Storrs CT USA

**Keywords:** *Caenorhabditis elegans*, fat accumulation, inverse molecular docking, thymol

## Abstract

As a natural product isolated from thyme oil in *thyme*, thymol (2‐isopropyl‐5‐methylphenol) harbors antiviral, antioxidant, and other properties, and thus could be potentially used for the treatment of various diseases. However, the function of thymol has not been comprehensively studied. Here, we applied an inverse molecular docking approach to identify unappreciated functions of thymol. Potential targets of thymol in humans were identified by the server of DRAR‐CPI, and targets of interest were then assessed by GO and KEGG pathway analysis. Subsequently, homologous proteins of these targets in *Caenorhabditis elegans* were identified by Blast tool, and their three‐dimensional structures were achieved using Swiss‐Model workspace. Interaction between thymol and the targeted proteins in worms was verified using AutoDock 4.0. Analyses of the targets revealed that thymol could be potentially involved in the glycolysis/gluconeogenesis and fatty acid degradation pathways. To verify the activity of thymol on lipid deposition in vivo, the *C*. *elegans* model was established. The lipid content of nematodes induced by high‐dose glucose was determined by Oil Red O and Nile Red staining, and gene expression was assessed by qRT‐PCR. The results showed that thymol might lead to the acceleration of β‐oxidation by upregulating *cpt‐1*, *aco*, *fabp,* and *tph‐1*, causing the descent of lipid content in nematodes. Our findings indicated that thymol could be potentially used for the treatment of chronic metabolic diseases associated with increased fatty acid deposition.

## Graphical abstract

In this study, we found a novel function of thymol on the inhibition of fat deposition induced by high‐dose glucose in Caenorhabditis elegans based on bioinformatics prediction combined with experimental verification in vivo. Our data suggested that only 1 mg/ml thymol significantly alleviated lipid accumulation induced by 1 mM glucose. Further qPCR analysis revealed that thymol might lead to the acceleration of β‐oxidation by upregulating cpt‐1, aco, fabp, and tph‐1, causing the fall of lipid content in nematodes. Additionally, bioinformatics analysis supported a suspicion about acceleration of β‐oxidation.

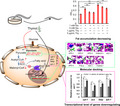

## 1 INTRODUCTION

Metabolic diseases, such as obesity and type‐2 diabetes, have been becoming severe health problems. Obesity is considered to be the result of multiple genetic factors and environmental influences. In modern society, foods and drinks rich in high energy including fat and sugar can be seen everywhere. A high‐fat and high‐sugar diet has become the main diet structure at present and is considered as one of the main causes of obesity caused by the environment.

However, sugar‐induced obesity is different from fat‐induced obesity. On the one hand, sugar can inhibit fat degradation; on the other hand, it also can be oxidized and decarboxylated to acetyl‐CoA that is the material for the synthesis of fatty acid. As we have known that glycolysis glucose is metabolized to acetyl‐CoA from pyruvate by pyruvate dehydrogenase under aerobic conditions, and acetyl‐CoA is the substrate of lipid biosynthesis. The rising blood glucose level triggers the release of insulin, leading to the dephosphorylation and activation of acetyl‐CoA carboxylase (ACC) by insulin‐dependent protein phosphatase. ACC catalyzes the formation of the first intermediate “malonyl‐CoA” of fatty acid synthesis, which suppresses carnitine acyltransferase I, thereby preventing fatty acid entry into the mitochondrial matrix for degradation through β‐oxidation (Zheng & Greenway, 2012).

Recently, more national authorities and the World Health Organization (WHO) have provided advice on daily diets urging a limited consumption of rapidly digestible starches and sugars (Brouns, 2018). A low‐energy diet and regular physical exercise are generally considered safe ways to prevent obesity. Besides, star molecular conjugated linoleic acid (CLA) has been widely applied to adjuvant therapy for chronic metabolic disease. CLA plays a role in the reduction of lipids storage, synthesis, and adipogenesis in adipocytes, and the enhanced fat utilization in muscle via fatty acid β‐oxidation (Park & Pariza, 2007). However, some researchers have expressed concerns regarding the safety of CLA for humans (Kelley & Erickson, 2003; Larsen et al., 2003; Riserus et al., 2002; Tricon & Yaqoob, 2006). The main concerns were the involvement of CLA in fatty liver, lipodystrophy, oxidative stress, and glucose intolerance (Clement et al., 2002; Pariza, 2004; Poirier et al., 2005; Tricon & Yaqoob, 2006). Therefore, it is essential to identify alternatives to interfere with fat deposition.

Natural essential oil constituents present promising potential to treat infection (Zhang et al., 2018), cancer (Xie et al., 2017), chronic inflammation (Yao et al., 2018), and even Alzheimer's disease (Postu et al., 2019). These constituents cover a group of biologically active molecules, including monoterpenes, sesquiterpenes, oxygenated monoterpenes, oxygenated sesquiterpenes, and phenolics among others. As one of the major constituents of essential oil in *Monarda punctate*, thymol (2‐isopropyl‐5‐methylphenol) has antimicrobial (Marchese et al., 2016), antioxidant (Luna et al., 2017), antiviral (Lai et al., 2012), antitumor (Gunes‐Bayir et al., 2019), and anti‐inflamatory (Yao et al., 2018) properties. More importantly, evaluations from the Joint FAO/WHO Expert Committee on Food Additives (JECFA) have indicated that there are no safety concerns on thymol in terms of acceptable daily intake (ADI). Although multiple functions of thymol have been revealed, a comprehensive analysis of thymol's functionality has not been conducted. Computation methodologies for the repositioning of drug molecules onto their receptors have been extensively utilized to reveal novel functions of small chemical compounds. Through reverse screening, we found that thymol may be involved in the regulation of obesity. At present, studies on the effect of thymol on obesity are few and mainly focus on lipid‐induced obesity. The effect of thymol on sugar‐induced obesity has not been studied. This study will start from this point, and further through the analysis of the glucose metabolism pathway to find the potential target of the action of thymol.

## 2 MATERIALS AND METHODS

### 2.1 Strains and chemicals

The wild‐type *Caenorhabditis* *elegans* strain N2 was obtained from the *C. elegans* Genetics Center (University of Minnesota, Minneapolis, MN, USA). The eggs of worms were incubated on nematode growth medium (NGM) agar plates at 20°C and seeded with a live *Escherichia coli* OP50 (*E. coli* OP50) as food resources.

### 2.2 Inverse molecular docking of thymol with human proteins

DRAR‐CPI server, a tool of inverse molecular docking, was developed to predict the drug target based on structural characteristics of protein (Luo et al., 2011). According to the algorithm in molecular docking program DOCK, the potential binding sites of receptor molecule in DRAR‐CPI are defined by overlapping spheres, consequently, achieving the reverse docking of the drug with these binding sites. Affinity for interaction energy in ligand receptors is evaluated by scoring function and then ranked to predict the potential targets. In this server, potential targets of a ligand can be obtained by submitting a mol2 formatted ligand file. In our study, the mol2‐format thymol file was downloaded from the following ZINC online website: (https://zinc.docking.org/substance/1529592).

### 2.3 Functional analysis for target genes

A PPI network was constructed to reveal the hub genes of the potential target proteins on STRING (https://string‐db.org/cgi/input.pl), a web portal for undermining the integrated function of multiple genes (Szklarczyk et al., 2015). Besides, on STRING, Gene Ontology (GO) enrichment tool including biological processes (BPs), cellular components (CCs), and molecular functions (MFs) was used to perform functional analysis of target proteins. Similarly, the Kyoto Encyclopedia of Gene and Genomes (KEGG) enrichment tool was used to analyze biological pathway enrichment to identify those pathways that responded to thymol. It should be noted that the protein name achieved from DRAR‐CPI needs to be changed to a discernible gene name by UniProtKB before submitting to STRING. The correlation degree for the proteins of interests was analyzed by MCODE, a Cytoscape plugin that can be used to identify clusters (highly interconnected regions) in a network.

### 2.4 Blast with Caenorhabditis elegans proteins and homologous modeling

Proteins associated with Glycolysis/Gluconeogenesis and fatty acid degradation pathway were picked, and their homologs in nematodes were identified by Blast analysis (https://blast.ncbi.nlm.nih.gov/Blast.cgi) (Hu et al., 2018). Three‐dimensional structures of these homologs were obtained through homology modeling using SWISS‐MODEL (https://www.swissmodel.expasy.org/) (Biasini et al., 2014).

### 2.5 Analysis of inverse docking and targeting sites prediction

Inverse docking was analyzed by Autodock 4.0, including AutoGrid and AutoDock. AutoGrid calculates related energy in a grid containing Van der Waals force, electrostatic force, and hydrogen bond force, while AutoDock conducts conformational search and evaluation using the Lamarckian Genetic Algorithm. For example, the docking of thymol with GAPDH could be realized by the following steps. The first step is the preparation of the system's coordinate files (PDBQT file) of GAPDH and thymol, including the removal of H_2_O (Eliminate the interference of solvation effect) and the addition of hydrogens (X‐ray diffraction technology cannot obtain the coordinate data for H) in GAPDH molecule, and the setting of active keys number in thymol. The second step is the setting of search sites, mainly including the adjustment of grid size in “Grad Box.” The third step is the establishment and operation of Autogrid parameter file with GPF format for thymol, generating GLG file and a series of MAP files of GAPDH that contain information of van der Waals forces, electrostatic forces, and dissolvent forces of different atom probe et al. The fourth step is the construction and running of Autodock parameter file with DPF format for thymol. Finally, we will achieve a DLG file of thymol. Docking files of GAPDH and thymol should be prepared for Pymol analysis, including the selection of thymol with the lowest energy, the sequence matching for GAPDH and thymol in Notepad++. In the end, a PDB file from Notepad++ needs to be opened in Pymol for the visualization and analysis of GAPDH‐thymol docking. We can adjust the appearance of their conformation and search targeting site of thymol by observing its hydrogen bonding site in Pymol.

### 2.6 Fat staining in *Caenorhabditis elegans*

Worms at the characteristic L4 larvae stage were chosen for synchronization. The procedure for glucose induction treatment was carried out as described previously (Zhao, 2015). Briefly, 1 mM and 5 mM glucose were added to the 50°C molten NGM medium. To determine the effect of thymol on lipid deposition, 1 μg/ml and 5 μg/ml of thymol were added to the NGM medium. The lipid content in the nematodes was evaluated by Oil Red O (ORO) (sigma) staining as described previously (Zhao, 2015). *Caenorhabditis elegans* at L4s were permeabilized through fixation, freezing and thawing, and dehydration process. Permeabilized worms were stained with ORO in the dark overnight at 1.07 *g*, 28°C. Decolorization of nematodes was then performed with 1, 2‐propylene glycol and washed with PBS buffer. The retention of ORO in nematodes could be observed with a 5× anatomical lens. The quantitative analysis was carried by the method of elution by ethanol. Specifically, quantified nematodes were eluted with absolute ethyl alcohol after discarding PBS buffer by centrifugation, and the collected supernatant was transferred to a 96‐well plate to measure the adsorption of ORO at 510 nm using a microreader.

Nile Red (Sigma) staining was also performed as described (Fouad et al., 2017). Briefly, 0.5 ml of the freshly diluted Nile Red (1 μg/ml) was added to plates seeded with *E.coli* OP50 and allowed to equilibrate for a minimum of 2 hr. Then, L1s obtained from bleaching were transferred to the plates and allowed to grow for the desired length of time. Stained nematodes were imaged under a 10× objective under a dark field and red fluorescence illumination with an inverted fluorescent microscope.

### 2.7 RNA isolation and qRT‐PCR

The total RNA of nematodes was isolated using TRIzol^®^ Reagent (Invitrogen) and purified with DNase I, RNase‐free (Thermo Scientific™). cDNA was obtained using M‐MuLV First Strand cDNA Synthesis Kit (Sangon). Real‐time PCR was performed using SYBR GreenⅠprobes by QuantStudio^®^ 3 System (Peng et al., 2016). Primer sequences were listed in Table S1. Relative expression of genes was determined using the 2‐ΔΔCt method and act‐1 was chosen as the reference gene.

### 2.8 Statistics analysis

All assays were repeated independently at least 3 times, and the experimental data were represented as mean ± *SD*. The data between two different treatments were compared statistically by one‐way ANOVA in SPSS. The significance of differences was defined at the *p* < .05 level and *p* < .01.

## 3 RESULTS

### 3.1 Protein targets prediction and analysis

**FIGURE 1:**
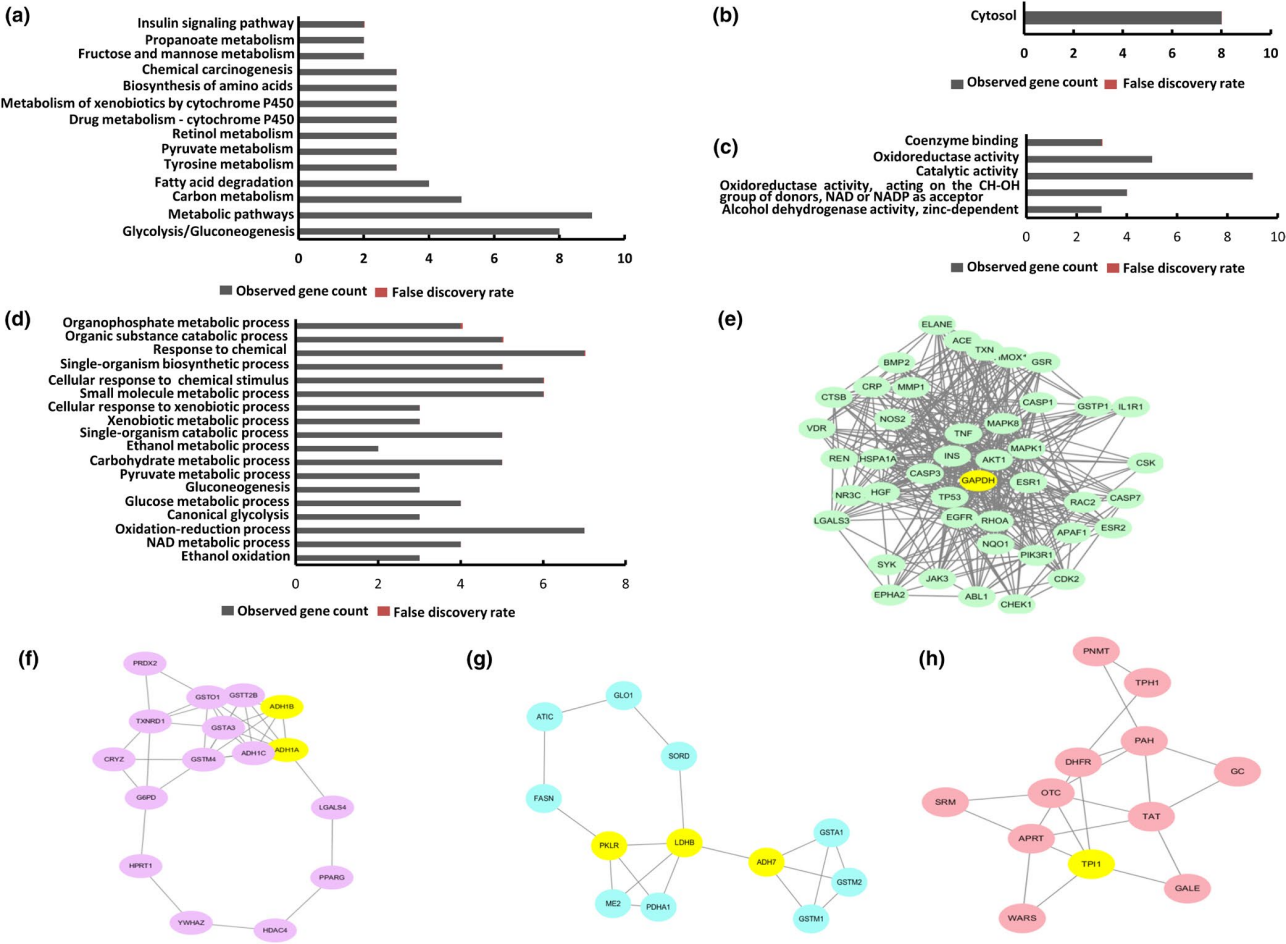
GO and KEGG pathway analysis of potential targets inferred to fat synthesis and metabolism. (a) KEGG pathway, (b) Cellular components pathway, (c) Molecular function pathway, (d) biological process pathway. (e–h) MCODE of proteins of interest in the whole PPI network

Analysis by DRAR‐CPI servers revealed that 392 human proteins have potential interaction with thymol. GO and KEGG analyses indicated that these targets of interest in glycolysis/gluconeogenesis and fatty acid degradation pathway were associated with carbon metabolism, tyrosine metabolism, pyruvate metabolism, retinol metabolism, drug metabolism‐cytochrome p450, metabolism of xenobiotics by cytochrome p450, biosynthesis of amino acids and chemical carcinogenesis pathways (Figure 1a). Furthermore, in GO enrichment, these targets were mainly localized in the cytosol (Figure 1b) and were associated with coenzyme binding, oxidoreductase activity, catalytic activity, oxidoreductase activity, acting on the CH‐OH group of donors, NAD or NADP as acceptor and alcohol dehydrogenase activity, zinc‐dependent (Figure 1c), and involved in organic substance catabolic process, single‐organism biosynthetic process, small molecule metabolic process, xenobiotic process, ethanol metabolic process, pyruvate metabolic process, glucose metabolic process, oxidation–reduction process, and ethanol process (Figure 1d).

Our analysis also showed that module 1 (Figure 1e) was important in the whole PPI network, with the highest score of 19.714. GAPDH (Glyceraldehyde‐3‐phosphate dehydrogenase) is a node in module 1 with the MCODE—score of 12.72, which has the highest complexity in 12 proteins responding to thymol, predicting it may be the central protein in glycolysis. Independent of its glycolytic activity, the encoded protein has additionally been identified to have uracil DNA glycosylase activity in the nucleus (Meyer‐Siegler et al., 1991). Also, GAPDH harbors a region that confers antimicrobial activity against *Staphylococcus bacteria* (Ong et al., 2019). Studies of a similar protein in mice have assigned a variety of additional functions including nitrosylation of nuclear proteins, the regulation of mRNA stability, and acting as a transferrin receptor on the cell surface of macrophage (Kornberg et al., 2010; Raje et al., 2007; Rodriguez‐Pascual et al., 2008). Module 2 (Figure 1f) had a score of 4.939 and included ADH1A and ADH1B nodes with the MCODE–score of 11, respectively. Module 3 (Figure 1g) had a score of 3.636 and was involved in the TPI note. Module 4 had a score of 3.273 and included three nodes involved in it including PKLR, LDHB, and ADH7 (Figure 1h). Besides, two other proteins (ACAT1 and FBP1) were not present in the module. The functional annotation of these representative proteins and reported experimental correlation with thymol were described in Table 1.

**TABLE 1:** Function annotation of potential targets of thymol

PDB ID	Protein name	Predicted docking score	Protein function and reported connection with diseases	Reported experimental correlation with thymol
1ZNQ	Glyceraldehyde‐3‐phosphate dehydrogenase	−22.3993	Catalyze the reversible oxidative phosphorylation in the presence of inorganic phosphate and nicotinamide adenine dinucleotide (NAD) in carbohydrate metabolism. Suppress proliferation and invasion of lung and esophageal squamous cell carcinomas (Hao et al., 2015)	No
1HSO	Alcohol dehydrogenase 1A	−23.3497	Catalyze the oxidation of alcohols to aldehydes. Variant confers susceptibility to esophageal squamous cell carcinoma (Cui et al., 2018). Polymorphisms in ADH1B associated with the increased risk of gastric cancer (Ghosh et al., 2017).	No
1HSZ	Alcohol dehydrogenase 1B	−22.79	Same as ADH1A	No
1D1T	Alcohol dehydrogenase class 4 mu/sigma chain	−25.5514	Same as ADH1A and ADH1B, however, the enzyme is inefficient in ethanol oxidation, but it is the most active as a retinol dehydrogenase, thus it may participate in the synthesis of retinoic acid, a hormone important for cellular differentiation. A single nucleotide polymorphism ADH7 is associated with the risk of squamous cell carcinoma of the head and neck (Wei et al., 2010).	No
1I0Z	L‐lactate dehydrogenase B chain	−25.1132	Catalyze the interconversion of pyruvate and lactate with concomitant interconversion of NADH and NAD^+^ in a post‐glycolysis process. Correlate With unfavorable survival in hepatocellular carcinoma (Chen et al., 2015), and promotion of pancreatic cancer progression (Cui et al., 2015).	No
2VGB	Pyruvate kinase PKLR	−24.1893	Catalyze the transphosphorylation of phohsphoenolpyruvate into pyruvate and ATP, which is the rate‐limiting step of glycolysis. Correlate the promotion colorectal cancer liver colonization(Nguyen et al., 2016).	No
1HTI	Triosephosphate isomerase	−22.7411	Catalyze the isomerization of glyceraldehydes 3‐phosphate (G3P) and dihydroxy‐acetone phosphate (DHAP) in glycolysis and gluconeogenesis. Variant (Arg189Gln) causes neurologic deficits (Roland et al., 2019).	No
2F2S	Acetyl‐CoA acetyltransferase	−22.6297	ACAT1 catalyzes the reversible formation of acetoacetyl‐CoA from two molecules of acetyl‐CoA. As a therapeutic target for Alzheimer's disease (Shibuya et al., 2015). ACAT1 suppresses diet‐induced obesity (Huang et al., 2018).	No
1FTA	Fructose−1,6‐bisphosphatase 1	−21.4765	FBP−1 catalyzes the hydrolysis of fructose 1,6‐bisphosphate to fructose 6‐phosphate and inorganic phosphate. Opposes renal carcinoma progression (Li et al., 2014), and regulates obesity(Lin et al., 2019).	No

### 3.2 Molecular docking

To confirm the activity of thymol on lipid synthesis and metabolism in vivo, we chosen nematodes as experimental models due to their advantages such as a short cycle, a fast reproduction rate, and high homology with human genes. Homologous genes of thymol's targets in nematodes were identified by Blast analysis (https://blast.ncbi.nlm.nih.gov/Blast.cgi). These analyses revealed *GPD‐1*, *GPD‐2*, *GPD‐3*, *GPD‐4*, *H24K24.3*, *TPI‐1*, *PYK‐1*, *PYK‐2*, *1DH‐1*, *KAT‐1*, *T02G5.7,* and *FBP‐1* in nematodes. However, their 3D structures remain unknown except for TPI‐1. Therefore, we constructed the 3D structure of the remaining protein by homology modeling technology. Homology modeling is a technique that can build a 3D structure for proteins based on the 3D structure of a similar protein. A homology model can provide an alternative for subsequent receptor‐ligand analyses such as molecular docking.

**FIGURE 2:**
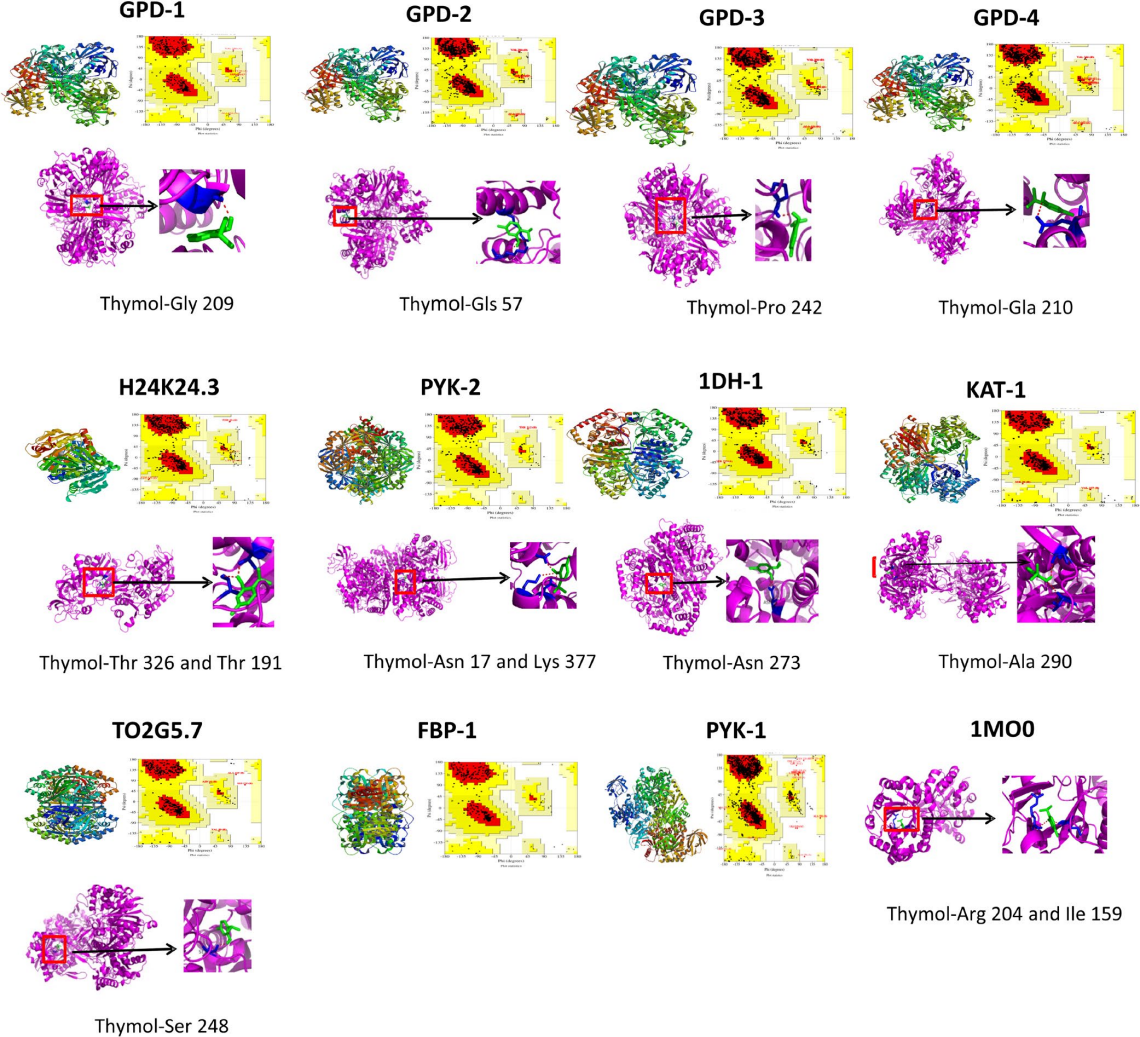
Homologous modeling of homologous proteins in nematodes at SWISS‐MODEL network, evaluation of their 3D structure by SAVES, and molecular docking between target proteins and thymol through AutoDock 4.0

In our study, we constructed models of these homology proteins by SWISS‐MOL and examined interactions between thymol and these proteins through Autodock 4.0 (Figure 2). Docking analysis revealed that thymol potentially interacts with the different amino acids of these proteins (except 1DH‐1) in nematodes (Table 2).

**TABLE 2:** Potential targets and corresponding amino acid action site of thymol

Potential targets	Corresponding amino acid action site	Three‐dimensional structure interaction diagrams
GPD‐1	Gly 209	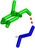
GPD‐2	Lys 57 and Arg 20	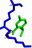
GPD‐3	Pro 242	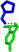
GPD‐4	Gln 210	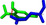
H24K24.3	Thr 326 and Thr 191	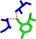
PYK‐2	Asn 17 and Lys 377	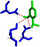
1DH‐1	Asn 273	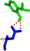
KAT‐1	Ala 290 and Val 27	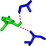
T02G5.7	Ser 248	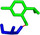
1MO0	Arg 204 and Ile 159	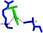

### 3.3 Thymol reduces fat deposition due to high glucose exposure in nematodes

**FIGURE 3:**
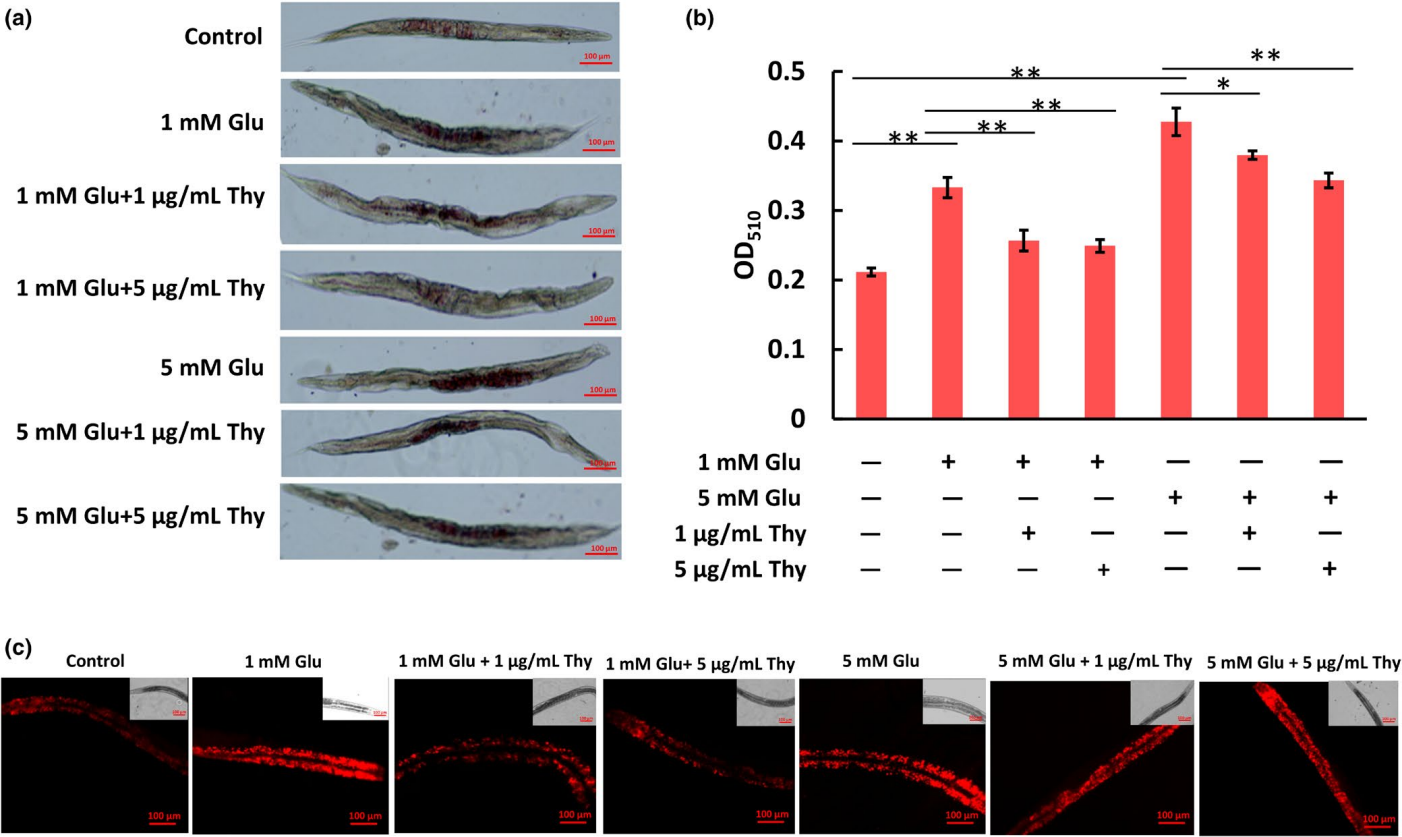
The effect of thymol on fat accumulation in nematodes induced by 1 mM and 5 mM glucose, respectively. (a) Fat deposition images of glucose‐induced nematodes postdifferent concentrations of thymol treatment and stained with Oil Red O. (b) Quantitative analysis of lipid accumulation in glucose‐induced nematodes treated with different concentrations of thymol and stained with Oil Red O through alcohol decolorization, *p < .05, **p < .01. (c) Fluorescent pictures of glucose‐induced nematodes treated with different concentrations of thymol and stained with Nile Red under an inverted fluorescent microscope

Fat accumulation induced by high‐dose glucose (1 mM or 5 mM) in nematodes is inheritable across several generations (Zhao, 2015). So we chose 1mM or 5mM of glucose to induce fat deposition in nematodes. ORO allows for the quantification of total lipid levels, while Nile red facilitates the evaluation of lipid distribution among tissues such as the hypodermis, intestine, and the germline. When used in conjunction, ORO and Nile Red enable the researchers to determine how genotype and environmental changes affect lipid accumulation and wherein the worm these changes are occurring (Wilber et al., 2017). The statistic result of ORO and Nile Red staining suggests both 1mM and 5mM of glucose are capable of inducing fat accumulation in the second generation of nematodes, and 5 mM‐induced fat accumulation is more significant than 1mM‐induced in the nematode (Figure 3b). Total lipid levels and lipid droplets were visualized by staining with ORO (Figure 3a) and Nile Red (Figure 3c), respectively. Further, the treatment of thymol can reduce lipid content. Importantly, 1 μg/ml and 5 μg/ml thymol treatment significantly reduced lipid deposition in glucose‐induced nematodes, indicating thymol can reduce fat content.

### 3.4 Thymol restores the change of transcriptional level of genes in β‐oxidation induced by high‐dose glucose

**FIGURE 4:**
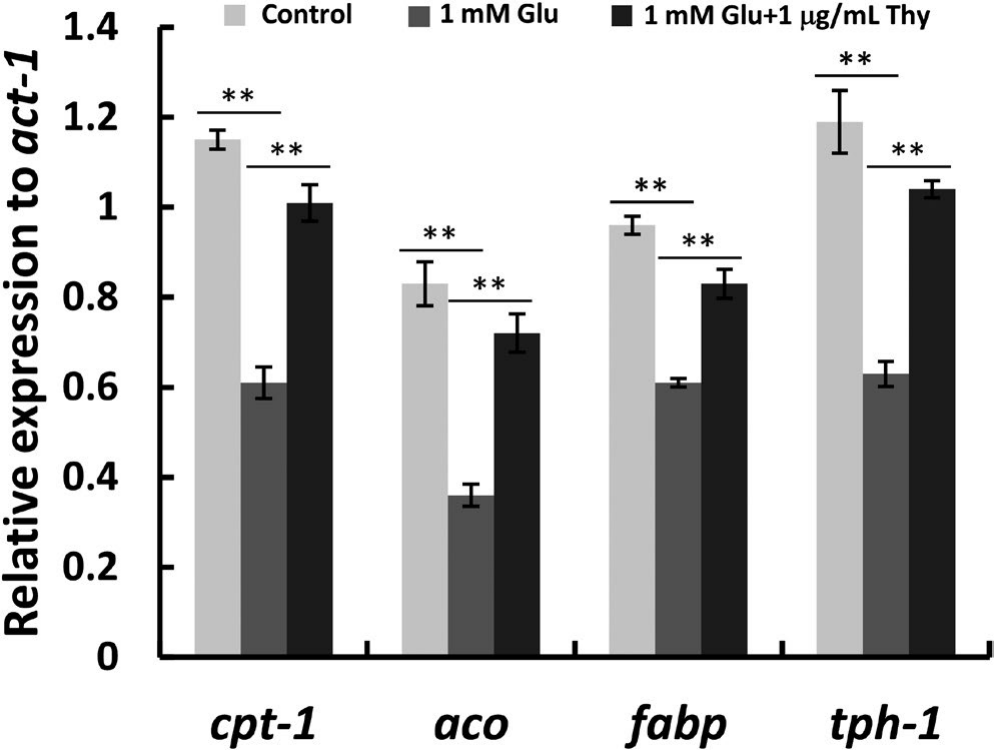
Transcriptional level of serotonin synthesis gene *tph‐1* and *cpt‐1*, *aco*, *fabp* in the process of β‐oxidation for the group of N2, glucose treatment, and thymol treatment, *p < .05, **p < .01

Our experimental data verified thymol could reduce lipid deposition in worms. We mentioned above that glucose‐induced fat accumulation is on the one hand due to the synthesis of acetyl‐CoA from glucose through glycolysis, which is the substrate of lipid biosynthesis, on the other hand, is due to the inhibition of fat degradation by the activation of acetyl‐CoA carboxylase (ACC) triggered by the rising blood glucose level. Therefore, we suspected that the reduction of fat accumulation by thymol might be achieved by regulating the upstream glycolysis pathway and downstream of fatty acid β oxidation. To confirm this idea, we determined the transcriptional level of key genes correlating with β‐oxidation including carnitine palmitoyltransferase (*cpt‐1*), acyl‐CoA oxidase (*aco*), fatty acid‐binding protein (*fabp*) in the process of β‐oxidation and tryptophan hydroxylase (*tph‐1*) in the upstream pathway of β‐oxidation. The result of qRT‐PCR suggested that the decreased level of *cpt‐1*, *aco*, *fabp,* and *tph‐1* in nematodes exposed by 1 mM glucose was upregulated after the exposure of thymol (Figure 4). These findings suggest the reduction of fat deposition caused by thymol is related to the oxidation of fatty acids.

## 4 DISCUSSION

In silico approaches can improve our ability to predict drug targets, thereby streamlining and accelerating the discovery process of a novel function of the drug. In this study, we found that thymol has a potential interaction with proteins associated with glycolysis/gluconeogenesis and fatty acid degradation pathway through reverse molecular docking approaches. These proteins included GAPDH, ADH1A, ADH1B, TPI‐1, PKLR, ADH7, LDHB, ACAT1, and FBP1 in humans. The homologous proteins in nematode are GPD‐1, GPD‐2, GPD‐3, GPD‐4, H24K24.3, TPI‐1, PYK‐1, PYK‐2, 1DH‐1, KAT‐1, T02G5.7, and FBP‐1, respectively. Some of the putative thymol targets have been identified as having an association with fat storage previously. For example, *kat‐1*, the major nematodes fat storage tissue, acts in the intestine. The gene transcription level of *kat‐1* was upregulated during fat feeding (Mak et al., 2006) and down‐regulated after treatment with the hesperidin (Peng et al., 2016). In nematodes, four genes designated *gpd‐1* through *gpd‐4* encode glyceraldehyde‐3‐phosphate dehydrogenase, and *△gpd‐1* can cause greatly raised triglycerides (Hegele et al., 2014). However, there is no known link between the remaining genes (including *pyk‐2, tpi‐1, 1dh1, to2g5.7, d2063.1, h24k24.3,* and *sodh‐2)* and obesity. These provide us with proof that this method has reliability for the prediction of thymol.

**FIGURE 5:**
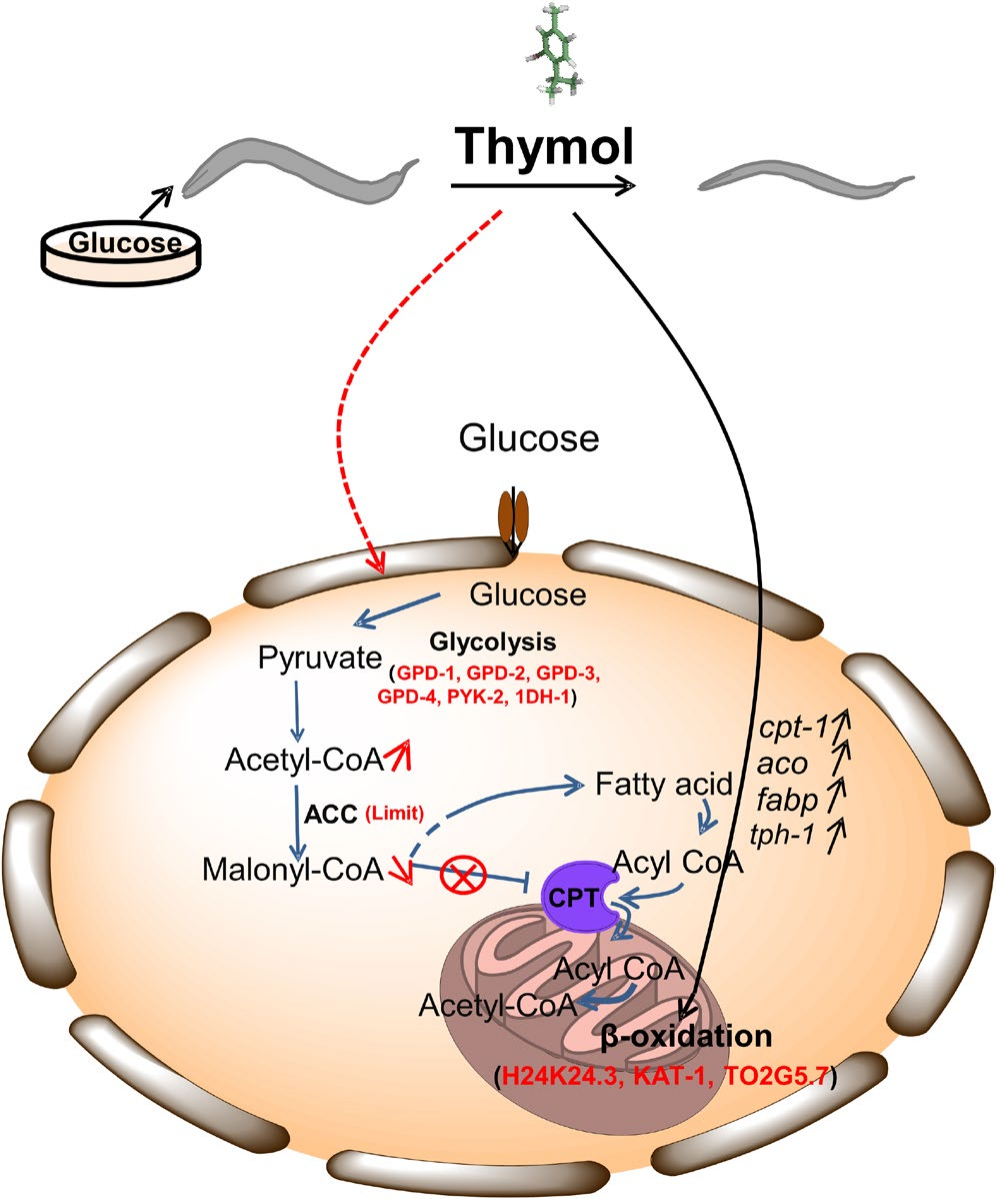
Diagram of thymol alleviating the obesity induced by glucose

Our experimental results in vivo verified that thymol inhibited lipid deposition induced by high‐dose glucose in nematodes. To understand how thymol makes a function in inhibiting fat accumulation, we detected the expression level of key genes correlation with β‐oxidation by qRT‐PCR. The findings suggested that thymol could recover the changes in the expression levels of these genes induced by high‐dose glucose, including *cpt‐1*, *aco*, *fabp,* and *tph‐1*. So thymol probably accelerates β‐oxidation according to the determination of qRT‐PCR combined with bioinformatics prediction. Furthermore, we suppose that thymol may accelerate glucose metabolism by regulating the targets of GPD‐1, GPD‐2, GPD‐3, GPD‐4, PYK‐2, 1DH‐1, and 1MO0 in the glycolytic pathway, causing the reduction of blood glucose level and ACC inactivation. ACC inhibition resulted in the reduction of malonyl‐CoA, which releases carnitine palmitoyltransferase (CPT) from inhibition and causes the transport of fatty acids into the mitochondria for β‐oxidation. And the protein targets we predicted including H24K24.3, KAT‐1, and T02G5.7 in the fatty acid degradation pathway may be located downstream of key genes correlating with β‐oxidation (Figure 5).

## CONFLICTS OF INTEREST

The authors declare no conflict of interest.

## AUTHOR CONTRIBUTIONS

**Fangfang Ban:** Data curation (equal); Formal analysis (equal); Investigation (equal); Methodology (equal); Software (equal); Validation (equal); Visualization (equal); Writing‐original draft (equal). **Liangbin Hu:** Conceptualization (equal); Funding acquisition (equal); Project administration (equal); Supervision (equal); Writing‐review & editing (equal). **Xiao‐Hui Zhou:** Conceptualization (equal); Writing‐review & editing (equal). **Yanyan Zhao:** Project administration (equal); Software (equal); Supervision (equal). **HaiZhen Mo:** Project administration (equal); Writing‐review & editing (equal). **Hongbo Li:** Project administration (equal); Supervision (equal); Writing‐review & editing (equal). **Wei Zhou:** Conceptualization (equal); Funding acquisition (equal); Project administration (equal); Writing‐review & editing (equal).

## ETHICAL STATEMENTS

This study does not involve any human or animal testing.

## Supporting information

Table S1
