# Supplementary material for: Inverse molecular docking reveals a novel function of thymol: Inhibition of fat deposition induced by high‐dose glucose in Caenorhabditis elegans
Source: Food Sci Nutr. 2021 Jun 11;9(8):4243–53. doi: 10.1002/fsn3.2392 (PMC8358335; doi:10.1002/fsn3.2392)
Supplement: Supplementary file 1 — Table S1 [file FSN3-9-4243-s001.docx]

Table S1 Primers for qRT-PCR analysis in the study

| Gene | Forward Sequences (5’-3’) | Reverse Sequences (5’-3’) |
| --- | --- | --- |
| *act-1*  *tph-1* | GCCGGAGACGACGCTCCACGCG  CGGTGAGCCAATTCCGCGAA | GCCTCGTCTCCGACGTACGAGTC  AGAAACTGCTTGCATGCGTGC |
| *cpt-1* | ACGGCATGGATCTCAAAGAC | GACGATCGACTCCTTGCCC |
| *aco* | ATATCTGGAGAAGATTCGACC | CAAGTACTTATCGACTGACGG |
| *fabp* | TTCTCGAGTTGAGAGATCGG | ATTCTGGCTCTCCGATCCG |
